# Zwitterionic coating assisted by dopamine with metal-phenolic networks loaded on titanium with improved biocompatibility and antibacterial property for artificial heart

**DOI:** 10.3389/fbioe.2023.1167340

**Published:** 2023-04-17

**Authors:** Lingwei Meng, Chuangxin Huang, Xin Liu, Hongyi Qu, Qiuliang Wang

**Affiliations:** ^1^ School of Rare Earth, University of Science and Technology of China, Hefei, China; ^2^ Ganjiang Innovation Academy, Chinese Academy of Science, Ganzhou, China; ^3^ Institute of Electrical Engineering, Chinese Academy of Science, Beijing, China

**Keywords:** artificial heart, titanium, Cu^2+^ initiator, co-deposition coatings, biocompatibility, antibacterial activity

## Abstract

**Introduction:** Titanium (Ti) and Ti-based alloy materials are commonly used to develop artificial hearts. To prevent bacterial infections and thrombus in patients with implanted artificial hearts, long-term prophylactic antibiotics and anti-thrombotic drugs are required, and this may lead to health complications. Therefore, the development of optimized antibacterial and antifouling surfaces for Ti-based substrate is especially critical when designing artificial heart implants.

**Methods:** In this study, polydopamine and poly-(sulfobetaine methacrylate) polymers were co-deposited to form a coating on the surface of Ti substrate, a process initiated by Cu^2+^ metal ions. The mechanism for the fabrication of the coating was investigated by coating thickness measurements as well as Ultraviolet–visible and X-ray Photoelectron (XPS) spectroscopy. Characterization of the coating was observed by optical imaging, scanning electron microscope (SEM), XPS, atomic force microscope (AFM), water contact angle and film thickness. In addition, antibacterial property of the coating was tested using *Escherichia coli* (*E. coli*) and *Staphylococcus aureus* (*S. aureus*) as model strains, while the material biocompatibility was assessed by the antiplatelet adhesion test using platelet-rich plasma and *in vitro* cytotoxicity tests using human umbilical vein endothelial cells and red blood cells.

**Results and discussion:** Optical imaging, SEM, XPS, AFM, water contact angle, and film thickness tests demonstrated that the coating was successfully deposited on the Ti substrate surface. The biocompatibility and antibacterial assays showed that the developed surface holds great potential for improving the antibacterial and antiplatelet adhesion properties of Ti-based heart implants.

## 1 Introduction

Heart failure is one of the most serious diseases threatening human life (Blecker et al., 2013; Lesyuk et al., 2018; Groenewegen et al., 2020; Heidenreich et al., 2022). Heart transplantation, drug therapy and artificial heart implantation are considered to be the main measures to treat heart failure until now (Crespo-Leiro et al., 2018). Due to the serious shortage of heart donors (Guyette et al., 2016; Wang et al., 2022), as well as the limitations of drug therapy (Kresoja et al., 2017) and xenotransplantation (Reardon, 2022; Cooper et al., 2022; Griffith et al., 2022), artificial heart has actually become the most potential way to treat heart failure (Almond et al., 2013; Goldstein et al., 2019; Kormos et al., 2019; Mehra et al., 2019; Shah et al., 2022). Inevitably, patients implanted with artificial hearts will face some serious infection problems (Kirklin et al., 2017; Chouirfa et al., 2019; Mulzer et al., 2020; Zhou et al., 2021), for example, the massive reproduction of bacteria represented by *E. coli* (*Escherichia coli*) and *S. aureus* (*Staphylococcus aureus*) on the surface of the implant materials (Chen et al., 2021; Zhu et al., 2022). Research showed that the proliferation of bacteria on the surface of the material is mainly divided into two stages, the initial adhesion is reversible, and the second stage of adhesion is almost irreversible. When the bacteria form a biofilm on the surface of the implant material, it is difficult to eradicate the bacteria by antibiotic treatment, surgical washing or debridement (Chouirfa et al., 2019). In addition, long-term ingestion of antibiotics by patients will also cause many complications and produce superbacteria with extremely strong drug resistance (Li and Webster, 2018; Kumar et al., 2019). Some studies had shown that one of the most effective methods to inhibit the initial adhesion of bacteria is to endow the surface of implant materials with antibacterial activity through surface modification (Chouirfa et al., 2019; Chen et al., 2021; Fu et al., 2021). The artificial heart is mainly made of titanium and its alloys, which have good biocompatibility due to their excellent biological inertia. However, titanium itself has almost no antibacterial ability and hydrophilicity, which is easy to make proteins, bacteria and blood platelets adhere (Yu et al., 2014). In addition to causing infection it may cause thrombus. Overall, it is clinically important and challenging to explore surface modification methods that can impart titanium and its alloys strong antibacterial and antiplatelet adhesion properties (Yu et al., 2014; Chouirfa et al., 2019; Zhang et al., 2022).

Compared with antibiotics, inorganic and organic antibacterial agents are not easy to develop drug resistance and have been widely used. Copper was recognized by the United States Environmental Protection Agency as the first metal antibacterial agent in 2008 (Vincent et al., 2018). As one of the main antibacterial agents in the agricultural field, copper has been used to treat various plant microbial infections. In addition, copper is also widely used to kill bacteria and other microorganisms in the fields of drinking water treatment, medical equipment and food processing (Saidin et al., 2021; Wang et al., 2021; Zhang et al., 2021). As described in previous studies, the Cu^2+^ will destroy the cell membrane of bacteria and solidify the protein structure (Dan et al., 2005; Chen et al., 2012). At the same time, the difference in the concentration of ions inside and outside the cell will destroy the transport of nutrients needed by bacteria (Zhu et al., 2022), leading to their death. However, the toxicity of copper at higher doses cannot be ignored (Ingle et al., 2014; Bartmanski et al., 2019). Excessive copper will damage kidneys, liver and other organs (Gaetke et al., 2014; Bartmanski et al., 2019). In addition, excessive Cu^2+^ can promote the formation of neurotoxic reactive oxygen species and accelerate amyloid-*β* aggregation, thus increasing the risk of Alzheimer’s disease (Du et al., 2021). It is disappointing that many studies of implant materials using copper as an antibacterial agent did not pay more attention to the toxicity of copper. Although copper can achieve good antibacterial activity at low cytotoxicity (Radovanovic et al., 2014), its release amount and toxicity are still important considerations.

Polydopamine (PDA) is a material derived from mussel adhesive proteins that can adhere strongly to the surface of almost any material. PDA has excellent adhesion, hydrophilicity, metal coordination and oxidation resistance due to its catechol and primary amine groups (Ryu et al., 2018; Li et al., 2021). Therefore, PDA is often used as an intermediate material to construct composite biomaterials together with antibacterial agents or superhydrophilic polymers such as zwitterionic polymers.

The molecular structures of zwitterionic polymers have positively and negatively charged groups, which can closely combine with water molecules to form a strong hydrous layer (Blackman et al., 2019). Therefore, the surface modification of a material with zwitterionic polymer can significantly improve the hydrophilicity of a material surface, thus improving the ability of the material surface to resist the adsorption of platelets, bacteria and non-specific proteins (Molino et al., 2018; Liu et al., 2022). In this paper, zwitterion polymer, poly (sulfobetaine methacrylate) (PSBMA), was used for surface modification.

**SCHEME 1:**
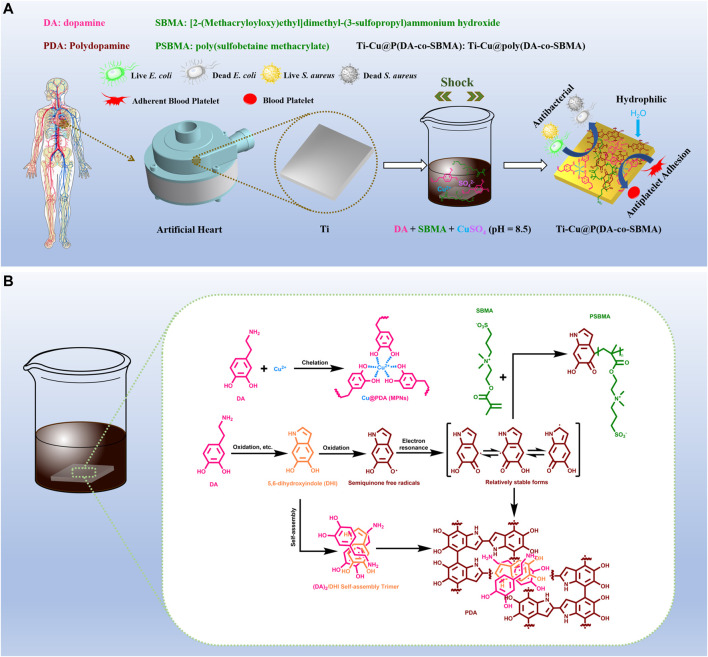
**(A)** Schematic diagram of the preparation process. **(B)** Possible mechanism for the reactions between Cu^2+^, DA and SBMA.

Based on the above description, it is concluded that Cu^2+^ is an agent with excellent antibacterial property, PDA has outstanding adhesion and metal coordination, and PSBMA possesses extreme hydrophilicity. Therefore, it is possible to use PDA as an intermediate material to anchor Cu^2+^ and PSBMA onto the surface of titanium. And it is worth noting that the standard redox potential of Cu^2+^/Cu is 0.34 V *versus* the normal hydrogen electrode (NHE), while that of dopaquinone/dopamine couple is close to 0.12 V *versus* NHE (Ball et al., 2013; Zhao et al., 2022), and the active free radicals produced by dopamine (DA) in the oxidation process can initiate the polymerization of zwitterionic monomers and anchor onto the surface of the material stably with zwitterionic polymers. It means that Cu^2+^ can be used as the oxidant and agglomerant to accelerate the oxidation and polymerization of dopamine, and the active free radicals generated during the oxidation of dopamine can initiate the polymerization of monomers of PSBMA, which together are firmly anchored to the surface of titanium to form a coating with strong antibacterial and antiplatelet adhesion properties. Cu^2+^ can be loaded on the PDA as cross-link sites, forming metal-phenolic networks (MPNs) (Yang et al., 2018), which enables the slow release of Cu^2+^ for a long time, thus ensuring long-lasting antibacterial capacity, low cytotoxicity and good stability of the coating (Liu et al., 2020). In this paper, CuSO_4_ was used to initiate the co-deposition of PDA and PSBMA on the surface of titanium. A functional coating with excellent antibacterial and antiplatelet adhesion properties was prepared successfully, as shown in Scheme 1A, which can effectively inhibit the proliferation of bacteria and improve the anti-fouling performance of titanium.

## 2 Materials and methods

### 2.1 Materials

Commercially pure titanium (99.7 wt%) was purchased from Shanghai Institute of Optics and Fine Mechanics, Chinese Academy of Science (Shanghai, China). [2-(methacryloyloxy)ethyl]dimethyl-(3-sulfopropyl)ammonium hydroxide”. There is only one space between “ammonium” and “hydroxide”, and no spaces between other words. (SBMA) was purchased from Sigma-Aldrich (USA). Potassium chloride (KCl, ≥99.5%) was obtained from Xilong Scientific Co., Ltd. (Guangdong, China). Copper sulfate pentahydrate (CuSO_4_⋅5H_2_O, 99.9% metals basis) was purchased from Shanghai Aladdin Biochemical Technology Co., Ltd. (Shanghai, China). DNA-Grade Tris–HCl solution (1 M), dopamine hydrochloride (DA HCl, 98%), sodium hydrogen phosphate dihydrate (Na_2_HPO_4_·2H_2_O, analytical reagent) and sodium chloride (NaCl, 99.99% metals basis) were purchased from Shanghai Macklin Biochemical Co., Ltd. (Shanghai, China), respectively. Potassium dihydrogen phosphate (KH_2_PO_4_, analytical reagent) was obtained from Sinopharm Chemical Reagent Co., Ltd. (Shanghai, China). *S. aureus* (ATCC29213), *E. coli* (ATCC25922) and blood were provided by Shiyanjia Lab (www.shiyanjia.com). human umbilical vein endothelial cells (HUVECs, iCell-h110) and the special culture medium for HUVECs (iCell-h110-001b) were purchased from iCell Bioscience Inc. (Shanghai, China). Other more detailed materials can be obtained from Supplementary Material.

### 2.2 Preparation of samples

Titanium plates were cut into rectangular samples with dimensions of 10 mm × 10 mm × 5 mm by a cutting machine. The titanium plates were first ground using 240 #, 1,000 #, and 2000 # sandpapers in succession and then polished to the mirror finish by nanoscale polishing liquid. Then they were ultrasonically cleaned with ultrapure water, acetone, and ethanol anhydrous for 30 min in succession, and then flushed with ultrapure water and dried in vacuum at 45°C, finally. These samples were labeled as pTi. After that, DA HCl, SBMA and CuSO_4_⋅5H_2_O were dissolved in 20 mL Tris–HCl solution (50 mM, pH = 8.5), and the concentrations of DA, SBMA, CuSO_4_ were kept at 2 mg/mL, 40 mg/mL and 0.8 mg/mL, respectively. The pTi samples were first immersed in the above solution at room temperature and shaken at 120 rpm for 12 h and then flushed with ultrapure water. Finally, the samples were dried in vacuum at 45°C for 4 h and labeled as Ti-Cu@poly(DA-co-SBMA) (Ti-Cu@P(DA-co-SBMA)). To compare the differences between different coatings, the single PDA coating (Ti-PDA), the PDA with PSBMA coating (Ti-P(DA-co-SBMA)) and the PDA coating with copper (Ti-Cu@PDA) were prepared on the surface of pTi using the same method in this work.

### 2.3 Characterization

The surface morphologies and surface roughness of samples were observed by a field emission scanning electron microscope (SEM, JSM-IT800, JEOL, Japan) and an atomic force microscope (AFM, Dimension Icon, Bruker, Germany) with a tapping probe (RTESPA-300, k = 40 N/m). An X-ray photoelectron spectroscopy (XPS, K-Alpha, Thermo Scientific, USA) was used to confirm the surface chemical compositions. The thickness of the coatings was measured by a spectroscopic ellipsometry (M-2000v, J. A. Woollam, USA). The hydrophilicity of the coatings was assessed by the contact angle system (DSAHT1600, KRUSS, Germany), using a 2-μL ultrapure water droplet at room temperature. The Ultraviolet–visible (UV–vis) absorption of reaction solutions was measured by an enhanced version of double beam UV–vis spectrophotometer (TU-1901, Shanghai Spectrum Analyzer, China).

### 2.4 Coating stability

To assess the stability of the coating, The Ti-Cu@P(DA-co-SBMA) samples were immersed in 4 mL phosphate-buffered saline (PBS) of PH = 5.5, PH = 7.2 and PH = 8.5, respectively, and shaken at 120 rpm under 37 °C for one weak. Then the absorbance of the above PBS solutions at 420 nm was measured by the UV–vis spectrophotometer on day 1, day 3, and day 7. The surface morphologies on day 7 were observed also to evaluate the structural stability of the Ti-Cu@P(DA-co-SBMA).

Moreover, the stability of the coating when exposed to air was investigated by monitoring the water contact angle for 1 week and observing surface morphology on day 7.

### 2.5 *In Vitro* antibacterial test

First, Luria-Bertani (LB) liquid medium, LB solid medium and bacterial (*E. coli* or *S. aureus*) suspension are prepared for use. Among them, the mediums need to be sterilized. Then, the bacterial suspension was diluted to 10^6^ CFU/mL with the liquid medium, after that, the sterilized samples were first placed in the culture dishes, and then 12.5 μL of the diluted bacterial suspension was dribbled evenly onto the test surfaces of the samples. After incubation at 37°C for 24 h, the bacterial suspension on the test surface of each sample was eluted down by ultrasonic processing with 1 mL of sterile PBS solution. The eluate was serially diluted 10 times for counting with sterile PBS solution, and then 100 μL of the diluted solution was inoculated on the solid medium and incubated at 37°C for 18 h. Finally, the antibacterial rate was calculated based on the multiplier of dilution and the number of colonies.

### 2.6 *In Vitro* platelet adhesion

The anticoagulant whole blood was centrifuged at 1,000 rpm for 10 min to obtain supernatant liquid, and the supernatant platelet-rich plasma (PRP) was added into a 1.5 mL centrifuge tube for testing platelet adhesion. All samples were put in PBS of PH = 7.2 at room temperature for 12 h to balance, and then incubated with PRP at 37°C for 120 min. At the same time, PRP was incubated with clean glass as a positive control for platelet adhesion. After incubation, the surfaces were washed gently with normal saline to remove the unadhered platelets, and then added 4% paraformaldehyde fix solution and fixed at room temperature for 10 min. After that, all surfaces were washed with normal saline three times, ultrapure water once, and dried naturally. Finally, the typical adhesion morphologies of platelets on all surfaces were observed by the laser three-dimensional imaging microscopy system (VK-X150K, KEYENCE, Japan), and the number of platelets adhesion in each visual field was recorded.

### 2.7 *In Vitro* hemolysis assay

The rabbit whole blood was centrifuged at 1,000 rpm for 10 min, then 0.2 mL of erythrocyte sediment was taken and added into a 1.5 mL centrifuge tube with 1.0 mL of normal saline. After centrifugation at 1,000 rpm for 10 min, the supernatant of the above mixture in the centrifuge tube was carefully sucked out, and then 0.25 mL of normal saline was added into the centrifuge tube to prepare red blood cells suspension (RBCs) for use. All samples were put into a 5 mL centrifuge tube with 1 mL of normal saline at 37°C for 30 min to balance, and then separately dripped with 20 μL RBCs into centrifuge tube and incubated at 37°C for 120 min. To have a better comparison, 20 μL RBCs was dripped into 1 mL normal saline as the negative control groups and 1 mL deionized water as the positive control groups, and then incubated at 37°C for 120 min. After incubation, the RBCs was centrifuged at 3,500 rpm for 5 min, and then the 0.2 mL of supernatant was extracted and transferred into a 96-well plate. Finally, the absorbance at 545 nm was measured with an microplate reader (Varioskan Flash, Thermo Scientific, USA).

The equation for hemolysis rate can be expressed as:

$$Hemolysis\;rate\;\left(\%\right)=\frac{D_{t}-D_{nc}}{D_{pc}-D_{nc}}\times 100\%$$

where *D*_t_, *D*_nc_ and *D*_pc_ are the absorbance of test groups, negative control groups and positive control groups, respectively.

### 2.8 *In Vitro* cytotoxicity and ions release behavior

The Cell Counting Kit-8 (CCK-8) and LIVE/DEAD cell staining methods were applied to evaluate the biocompatibility of the samples with HUVECs. In brief, there were 6 groups, the control group and the sample groups 1 ∼ 5. For the control group, 1 mL/well of cell suspension was added, and for the sample groups, the sterilized samples were placed in the wells first, and then 1 mL/well of cell suspension was added. Three replicate wells were made for each group. HUVEC cells at the logarithmic growth stage were taken for cell counting and seeded into 24-well plates by adjusting the cell concentration at 6 × 10^4^/well. The cells were incubated with samples in a constant temperature incubator at 37°C/5% CO_2_ for 24 h and 48 h, respectively. After that, the medium was removed and the wells were washed with PBS three times, and then 1 mL of medium containing 10% CCK-8 was added to each well and incubated at 37°C/5% CO_2_ for 2 h. Finally, the absorbance values at 450 nm were measured by a microplate reader (SPARK 10 M, TECAN, Switzerland). In addition, for LIVE/DEAD cell staining methods, the 24-well plates with cells cultured for 24 h or 48 h were added with Calcein-AM/PI dual staining solution, and then incubated for 15 min at room temperature and protected from light. Finally, the results were observed with an inverted fluorescence microscope (FV1200, Olympus, Japan).

Moreover, the release behavior of Cu^2+^ *in vitro* was investigated. The Ti-Cu@P(DA-co-SBMA) sample was immersed in 10 mL PBS of PH = 7.2, and shaken at 120 rpm under 37°C for one weak. Then, at 1, 3, 5, and 7 days, the concentration of Cu^2+^ was tested by the inductively coupled plasma optical emission spectrometer (ICP-OES, PQ 9000 Elite, Analytik Jena, Germany).

## 3 Results and discussion

### 3.1 Fabrication mechanism of Ti-Cu@P(DA-co-SBMA)

The polymerization process of DA has been shown to have many of the characteristics of a free radical process (Hong et al., 2012), but the more detailed mechanism is not fully understood (Dreyer et al., 2012; Liebscher et al., 2013). Scheme 1B shows the possible mechanism of the co-polymerization process of DA monomers and SBMA monomers and the formation of MPNs, the mechanism shown is based on previous studies (Hong et al., 2012; Ejima et al., 2013; Chen et al., 2021; Zhao et al., 2022). Under an alkaline condition (Hong et al., 2012), DA monomers undergo oxidation and a series of processes to form semiquinone free radicals. After being transformed into a relatively stable state *via* electron resonance (Chen et al., 2017), these semiquinone free radicals are coupled to form PDA, or initiate the polymerization of the SBMA monomers (Zhang et al., 2017; Ma et al., 2019). When CuSO_4_ is added, Cu^2+^ can participate in the oxidation and polymerization process of dopamine, including oxidizing dopamine with dissolved oxygen and stabilizing the structure of PDA as a cross-linking site to form MPNs.

**FIGURE 1:**
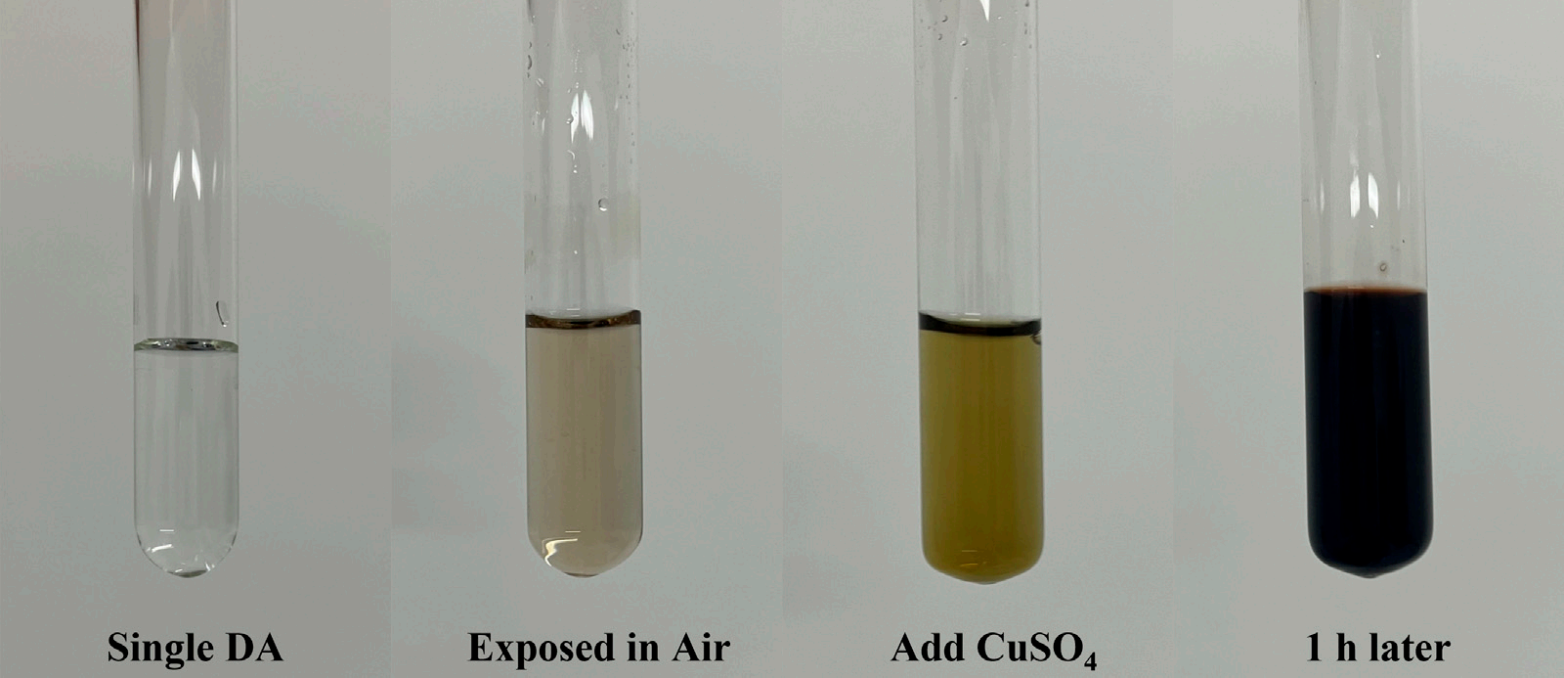
Photograph of the mixed solution under different conditions.

**FIGURE 2:**
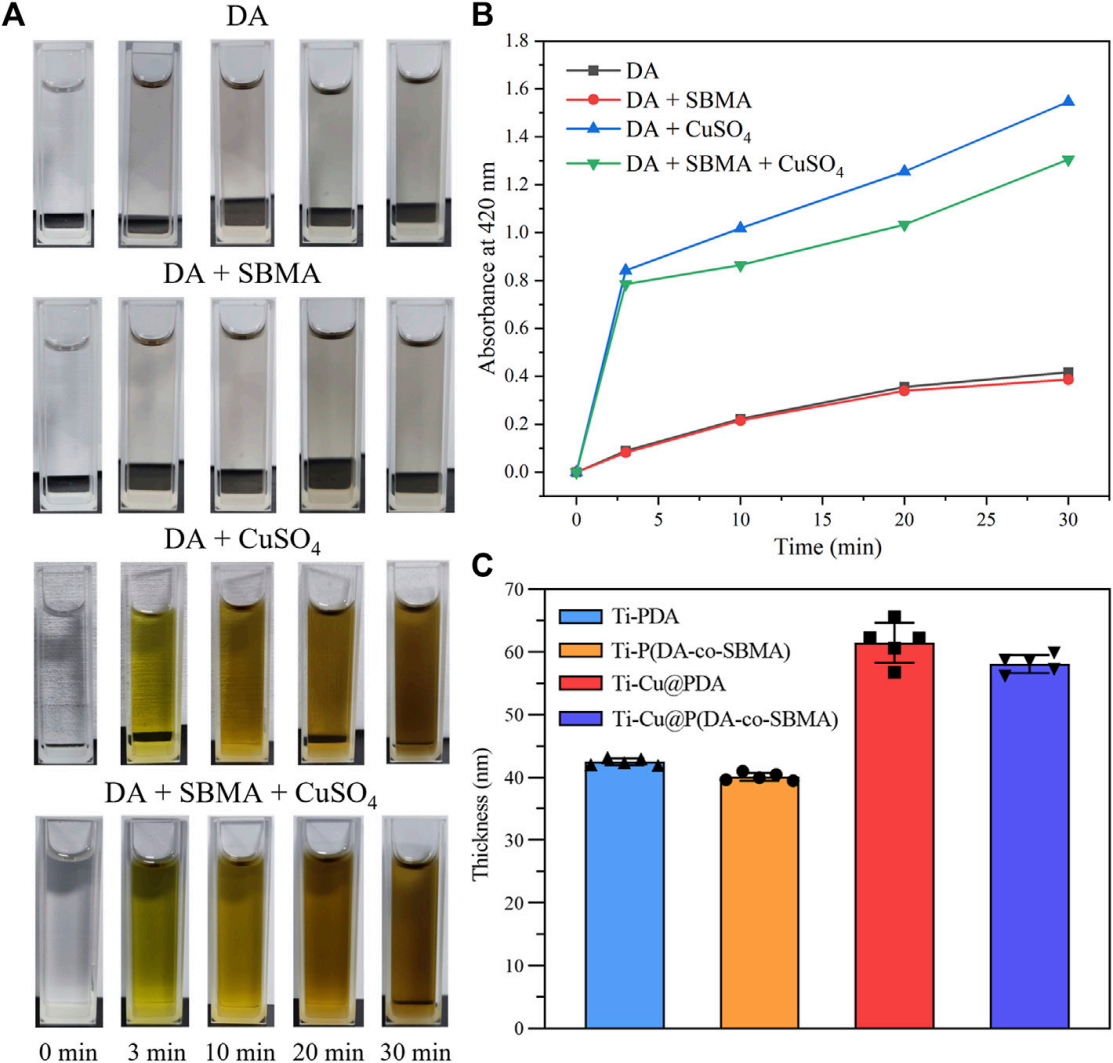
**(A)** Photographs of different solutions at different times. **(B)** UV–vis absorbance of different solutions at 420 nm with time. **(C)** The thickness of different coatings.

Figure 1 shows the color change of the different mixed solutions. After adding Tris–HCl solution (PH = 8.5) to dopamine solution and exposing to air for several minutes, the color of the solution rapidly changed from colorless to beige, indicating that dopamine is easy to be oxidized under alkaline conditions. After adding CuSO_4_, the color of the solution quickly changed to greenish brown. Finally, the solution turns dark brown after 1 h. This appearance shows that CuSO_4_ can greatly accelerate the oxidative polymerization process of dopamine. These conclusions can also be confirmed by the results in Figure 2. Figure 2A shows the color changes of different reaction solutions with time. It can be seen that the color of the solutions containing CuSO_4_ rapidly darkened and showed dark brown of PDA compared with DA and DA + SBMA systems. To further analyze the polymerization rate of DA in different systems, the UV–vis absorbance of different solutions at 420 nm with time was tested, and the results are shown in Figure 2B. The characteristic peak at 420 nm corresponds to the C=C−C=O structure of quinone from the polymerization of dopamine (Wang et al., 2017; Xiao et al., 2022). From the results of Figure 2B, it obviously indicated that CuSO_4_ can significantly accelerate the oxidative polymerization of DA in both the DA and DA + SBMA systems. CuCl_2_ and Cu(NO_3_)_2_ also exhibited the same acceleration effect (Supplementary Figure S1), which could prove that Cu^2+^, not SO_4_^2−^, accelerated the polymerization of DA. The absorbance of the solutions with SBMA at 420 nm presented a slower increase with time, confirming that SBMA monomers consume some quinones in the polymerization process and slow down the polymerization of PDA. The results of coatings thickness shown in Figure 2C show that Cu^2+^ significantly accelerated the growth of the coatings, while SBMA slightly slowed down the growth of the coatings, which agrees with the results of the above UV–vis absorbance test.

Briefly, these results confirm that DA is easily oxidized and rearranged to free radicals by dissolved oxygen or Cu^2+^ under alkaline conditions, and the polymerization of SBMA and DA is initiated by the free radicals.

### 3.2 Characterization of pTi and modified Ti

**FIGURE 3:**
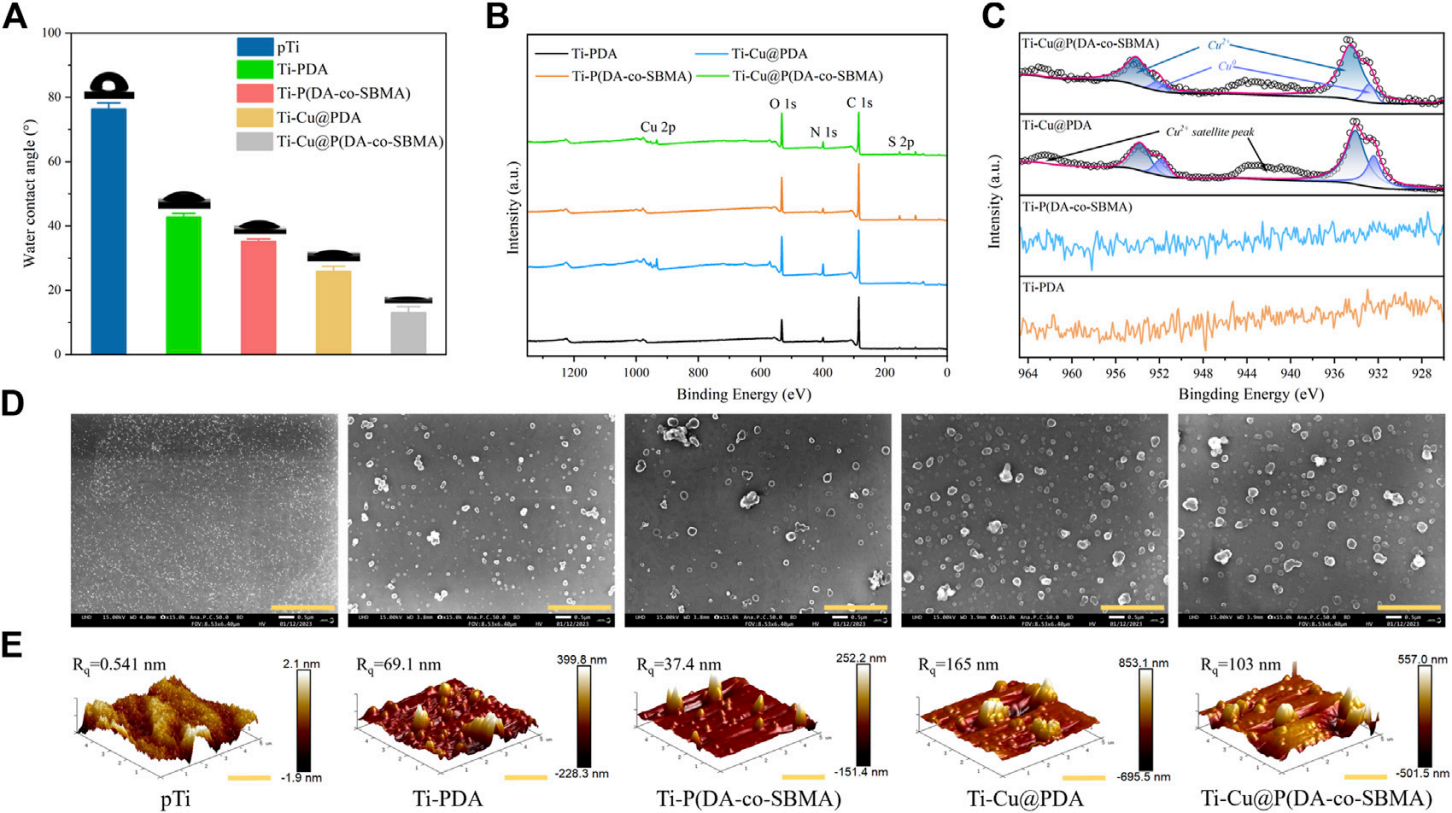
**(A)** Water contact angles of the pTi, Ti-PDA, Ti-P(DA-co-SBMA), Ti-Cu@PDA and Ti-Cu@P(DA-co-SBMA). **(B)** XPS spectra of the Ti-PDA, Ti-P(DA-co-SBMA), Ti-Cu@PDA and Ti-Cu@P(DA-co-SBMA). **(C)** High-resolution Cu 2p spectra of the Ti-PDA, Ti-P(DA-co-SBMA), Ti-Cu@PDA and Ti-Cu@P(DA-co-SBMA). **(D)** SEM images of the pTi, Ti-PDA, Ti-P(DA-co-SBMA), Ti-Cu@PDA and Ti-Cu@P(DA-co-SBMA). Scale bar: 2 μm. **(E)** AFM images of the pTi, Ti-PDA, Ti-P(DA-co-SBMA), Ti-Cu@PDA and Ti-Cu@P(DA-co-SBMA). Scale bar: 2 μm.

As Shown in Supplementary Figure S2, the color of the original pTi is silver gray, and that of Ti-Cu@P(DA-co-SBMA) is PDA-like brown, which initially indicates the successful preparation of the coating. The results of spectroscopic ellipsometry (Figure 2C) also shows that a nanoscale film was formed on the surface of the pTi. As shown in Figure 3A, the water contact angles of Ti-PDA and Ti-P(DA-co-SBMA) were obviously decreased compared with pTi, indicating that PDA and the PSBMA could significantly improved the hydrophilicity of material surface. Materials with good hydrophilicity can inhibit the adhesion of platelets and non-specific proteins and have excellent biocompatibility. Moreover, the hydrophilicity of the Ti-Cu@PDA and Ti-Cu@P(DA-co-SBMA) was even better, which could be attributed to the fact that Cu^2+^ can increase the thickness of coating in the same reaction time.

To further verify the success of the surface modification and research the characterizations of the coatings, XPS analysis was performed on the surfaces of different samples. Figure 3B shows the XPS spectra of Ti-PDA, Ti-P(DA-co-SBMA), Ti-Cu@PDA and Ti-Cu@P(DA-co-SBMA), showing the differences of the elements contained in each coating. The high-resolution N 1s spectra (Supplementary Figure S3) shows a slight shift of the N 1s peak near 402 eV to higher binding energy for Ti-P(DA-co-SBMA) and Ti-Cu@P(DA-co-SBMA) compared to that of Ti-PDA and Ti-Cu@PDA. Besides, for Ti-P(DA-co-SBMA) and Ti-Cu@P(DA-co-SBMA), the area proportion of the N 1s peak near 402 eV is higher. These could be ascribed to the N^+^ in C–N^+^ bond of PSBMA. Additionally, for Ti-P(DA-co-SBMA) and Ti-Cu@P(DA-co-SBMA), S 2p peak at 168.0 eV was observed (Supplementary Figure S4), which was attributed to the sulfonic acid group (–SO_3_H) of PSBMA. The high-resolution Cu 2p spectra (Figure 3C) for Ti-Cu@PDA and Ti-Cu@P(DA-co-SBMA) contained a strong Cu 2p3/2 peak (934.5 eV), a strong Cu 2p1/2 peak (954.1 eV) and two broad satellite peaks, attributed to the predominant presence of Cu^2+^, which could prove the formation of MPNs. In addition, a pair of weak peaks were detected at lower binding energy positions, presumably belonging to Cu^0^ reduced by dopamine. This could be further confirmed that Cu^2+^ is strong enough to oxidize dopamine.

The changes in surface morphology and roughness of the titanium sheet were observed by SEM and AFM. As shown in Figure 3D, the surface of the original pTi was smooth, and there were uniform and tiny TiO_2_ oxide particles, indicating that titanium is very easy to be oxidized. A large number of uniformly distributed particulate agglomerates were observed on the surface of Ti-PDA, Ti-P(DA-co-SBMA), Ti-Cu@PDA and Ti-Cu@P(DA-co-SBMA), which was consistent with the previous report (Ju et al., 2011; Chen et al., 2021), indicating the success of surface modification. Additionally, compared with Ti-PDA and Ti-P(DA-co-SBMA), the number and size of particulate agglomerates on the surface of Ti-Cu@PDA and Ti-Cu@P(DA-co-SBMA) were significantly increased, which was attributed to the acceleration of Cu^2+^ on the polymerization process of PDA. The decrease in the number of agglomerates in Ti-P(DA-co-SBMA) and Ti-Cu@P(DA-co-SBMA) could be ascribed to the consumption of some free radicals by SBMA. Similar appearance can also be found in the observation of AFM (Figure 3E). The AFM images show that after immersion, a mass of nano-particles were formed on the smooth titanium sheet surface and the surface roughness was increased significantly. In addition, the maximum vertical height of agglomerates and the root-mean-square roughness (Rq) of Ti-P(DA-co-SBMA) and Ti-Cu@P(DA-co-SBMA) was decreased compared with Ti-PDA and Ti-Cu@PDA, indicating that SBMA monomers competed with DA monomers for free radicals in the reaction and slowed the polymerization and deposition of PDA, on the other hand, that of Ti-Cu@PDA and Ti-Cu@P(DA-co-SBMA) was increased compared with Ti-PDA and Ti-P(DA-co-SBMA), this was due to the rapid oxidative polymerization of DA monomers in the CuSO_4_ system. These results are in good agreement with the conclusions obtained in the last section.

### 3.3 Coating stability of Ti-Cu@P(DA-co-SBMA)

**FIGURE 4:**
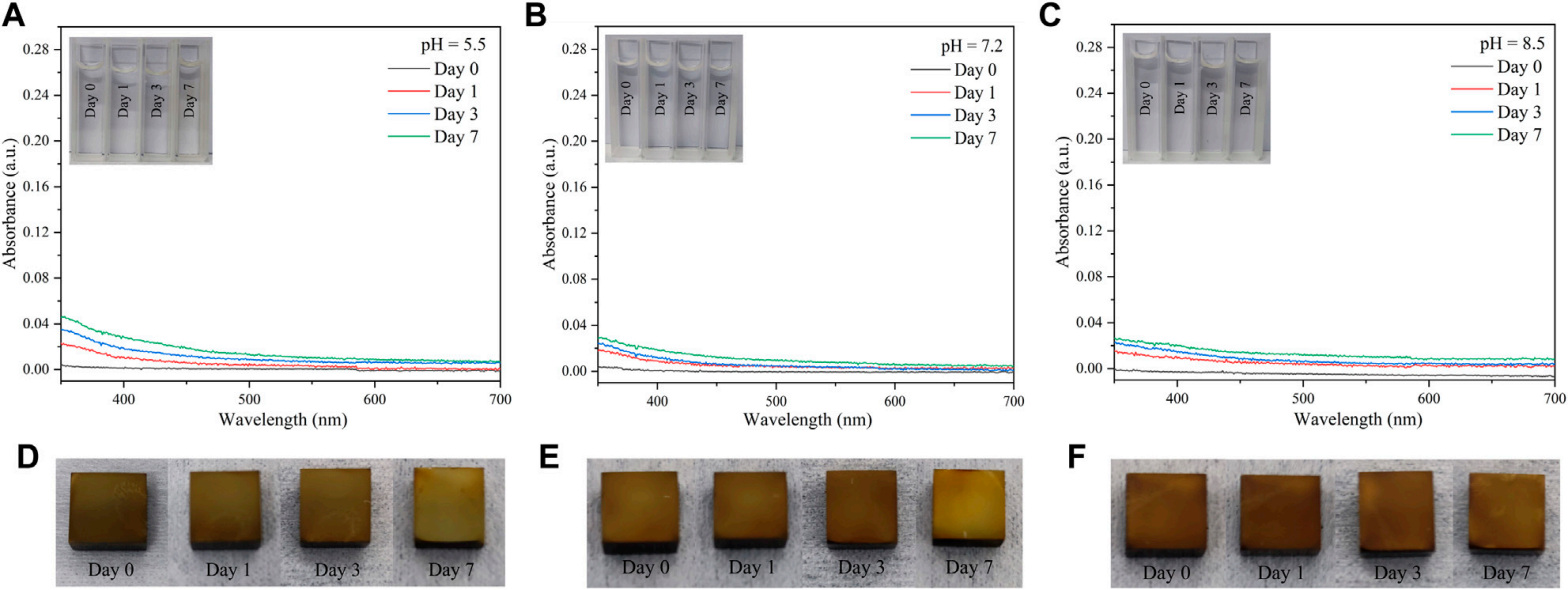
UV–vis spectra of different eluents [**(A)** pH = 5.5, **(B)** pH = 7.2, and **(C)** pH = 8.5] of Ti-Cu@P(DA-co-SBMA) samples after different days. Photos of Ti-Cu@P(DA-co-SBMA) samples immersed in different PBS buffer solutions [**(D)** pH = 5.5, **(E)** pH = 7.2, and **(F)** pH = 8.5] after different days.

As an implant material, the coating need to have good stability. To test the stability of the coating, the PDA content in different PBS solutions after immersing Ti-Cu@P(DA-co-SBMA) samples for some days was detected by UV–vis spectroscopy near 420 nm. Figures 4A–C show small changes in the UV–vis absorbance of the solutions, indicating that there was only a small amount of PDA detaching from the surfaces of Ti-Cu@P(DA-co-SBMA) in the solutions. The appearances of Ti-Cu@P(DA-co-SBMA) after immersion also changed slightly as shown in Figures 4D–F, and the surface morphologyies was also unchanged after a week (Supplementary Figure S5), showing the good stability of the coating when immersed in solutions with different pH. This could be attributed to the fact that Cu^2+^ could chelate with the amine and imine groups of PDA to stabilize the structure of PDA (Zhu et al., 2022).

**FIGURE 5:**
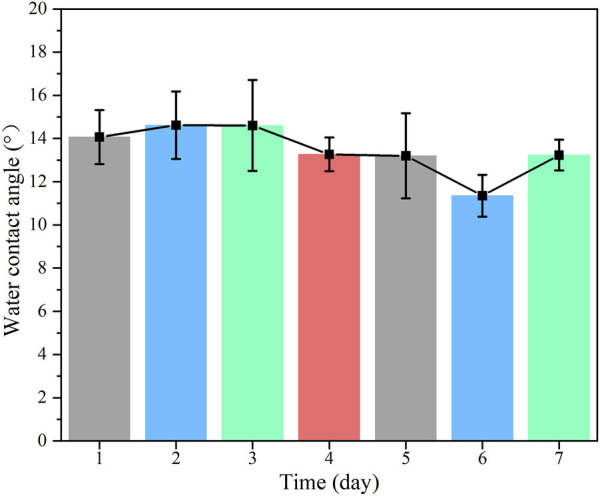
The change of water contact angle of the Ti-Cu@P(DA-co-SBMA) exposed to air for 7 days.

In addition, the stability of the coating exposed to air was also investigated by monitoring the water contact angle for 1 week. There was almost no change in the water contact angle (Figure 5). As shown in Supplementary Figure S6, the surface morphology was also unchanged after 1 week. These results all indicated the good stability of the coating when exposed to air.

### 3.4 *In Vitro* antibacterial activity

**FIGURE 6:**
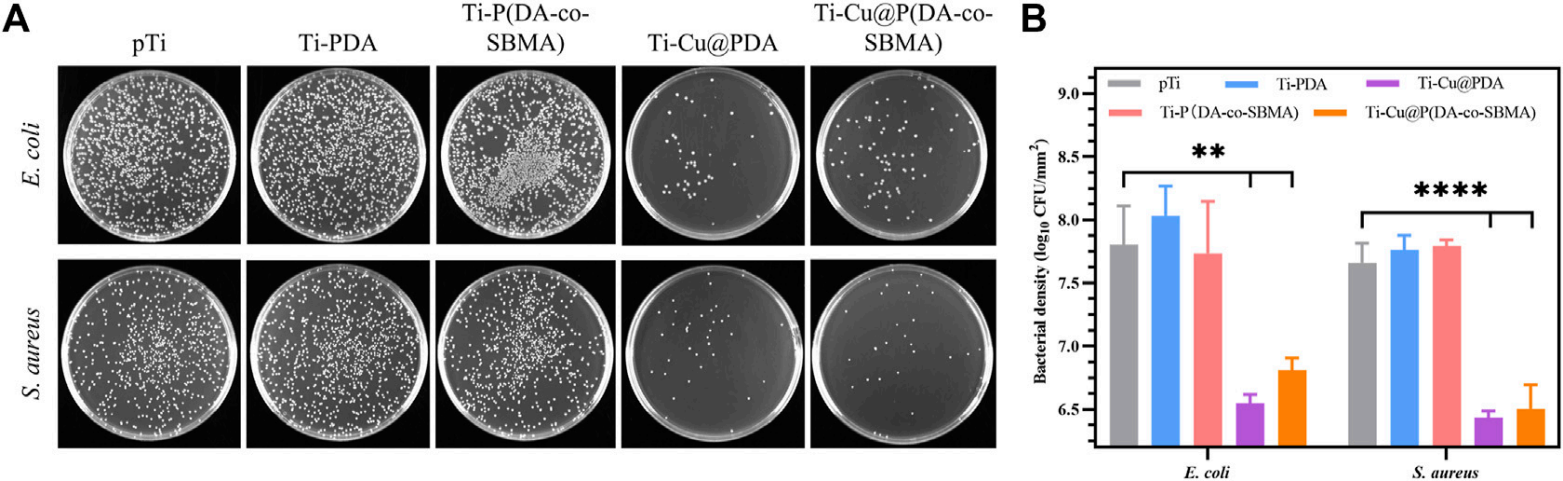
**(A)** Photographs and **(B)** bacterial numbers of *Escherichia coli* and *Staphylococcus aureus* in the mediums after incubation with pTi, Ti-PDA, Ti-P(DA-co-SBMA), Ti-Cu@PDA and Ti-Cu@P(DA-co-SBMA), respectively (**p < 0.01, ****p < 0.0001).

To explore the antibacterial properties of the materials, representative *E. coli* (Gram-negative bacteria) and *S. aureus* (Gram-positive bacteria) were selected for incubation on the surface of the samples. As shown in Figure 6A, compared to the other samples, the number of colonies of both bacteria was significantly reduced after incubation on the surfaces of Ti-Cu@PDA and Ti-Cu@P(DA-co-SBMA), showing strong antibacterial activity.

To further evaluate the antibacterial performance of the coatings containing copper, the number of bacterial colonies after incubation was counted. As shown in Figure 6B, the numbers of colonies of both bacteria on the surfaces of the samples modified with PDA or PDA and PSBMA were almost the same or even more than that of the primordial pTi. Some studies have reported that PDA polymerized in Tris–HCl solvent showed antibacterial activity in PBS and saline media (Patel et al., 2018), but its antibacterial ability cannot meet the conventional requirements, and in most cases, it needs to cooperate with other materials under special conditions such as light irradiation to have obvious antibacterial activity (Xu et al., 2022). In the medium containing a large amount of nutrients, PDA itself is more difficult to inhibit the mass reproduction of bacteria. Moreover, as shown in the AFM results (Figure 3E), the surface roughness of the coatings was far greater than that of pTi, which might lead to easier adhesion and reproduction of bacteria, because of the positive correlation between adhesion and reproduction of bacteria and surface roughness (Almaguer-Flores et al., 2012). And the hydrophilic (not superhydrophilic) surface might be difficult to resist the proliferation of bacteria under static culture condition. This appearance was consistent with that reported in (Ho and Ding, 2015). The numbers of both bacterial colonies were reduced by more than 1 log after incubation on the surfaces of Ti-Cu@PDA and Ti-Cu@P(DA-co-SBMA). The antibacterial rate of the coatings is greater than 90%. Additionally, the antibacterial properties of copper against the two bacteria were slightly different, which could be put down to the different structures of the cell walls of Gram-negative and Gram-positive bacteria (Epand et al., 2016; Saidin et al., 2021; Angane et al., 2022; Zhu et al., 2022).

### 3.5 Antiplatelet adhesion property

**FIGURE 7:**
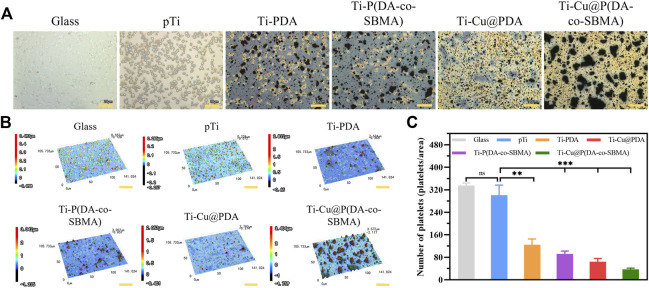
**(A)** optical images, (Scale bar: 20 μm) **(B)** 3D images (Scale bar: 30 μm) and **(C)** numbers of adhered platelets on the surfaces of Glass, pTi, Ti-PDA, Ti-P(DA-co-SBMA), Ti-Cu@PDA and Ti-Cu@P(DA-co-SBMA) (^*ns*^ *p* > 0.05, **p < 0.01, ***p < 0.001). The size of each observed area is 141.024 × 105.733 μm.

As shown in Figures 7A, B, platelets could adhere to the surfaces of glass (positive control) and pTi, and most of them stretched out pseudopodia, showing a typical activation state, which indicated that the function of the experimental platelets were normal and the method was effective. It can be seen from the figures, a large number of platelets adhered to the glass and the original pTi. In contrast, there were only a few platelets adhered on the surfaces of the samples after surface modification. It should be noted that the large agglomerates in the Figure 7B were coating particles. The statistical datas shown in Figure 7C more clearly show the difference in the number of platelets adhering to the surface of each sample. After surface modification, the ability of titanium to resist platelets adhesion was significantly improved, especially the platelets density on surface of Ti-Cu@P(DA-co-SBMA) was only 12.38% of that on surface of pTi, because of the higher content of PSBMA on its surface, indicating that the coating had excellent antiplatelet adhesion property.

### 3.6 *In vitro* toxicity

**FIGURE 8:**
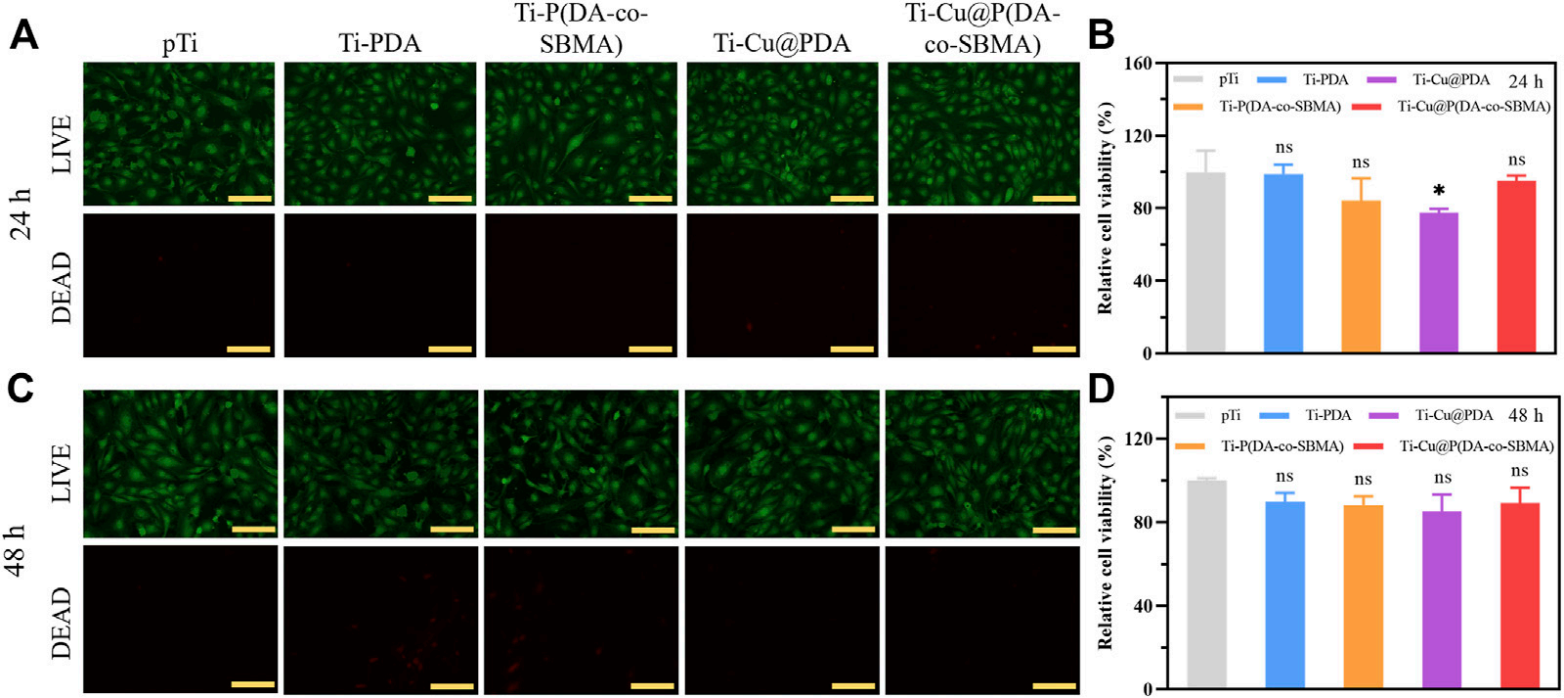
Fluorescent images of LIVE/DEAD staining of HUVECs after incubation with pTi, Ti-PDA, Ti-P(DA-co-SBMA), Ti-Cu@PDA and Ti-Cu@P(DA-co-SBMA) for **(A)** 24 h and **(C)** 48 h. Relative cell viability of HUVECs after incubation with pTi, Ti-PDA, Ti-P(DA-co-SBMA), Ti-Cu@PDA and Ti-Cu@P(DA-co-SBMA) for **(B)** 24 h and **(D)** 48 h (^*ns*^ *p* > 0.05, *p < 0.05). Scale bar: 150 μm.

**FIGURE 9:**
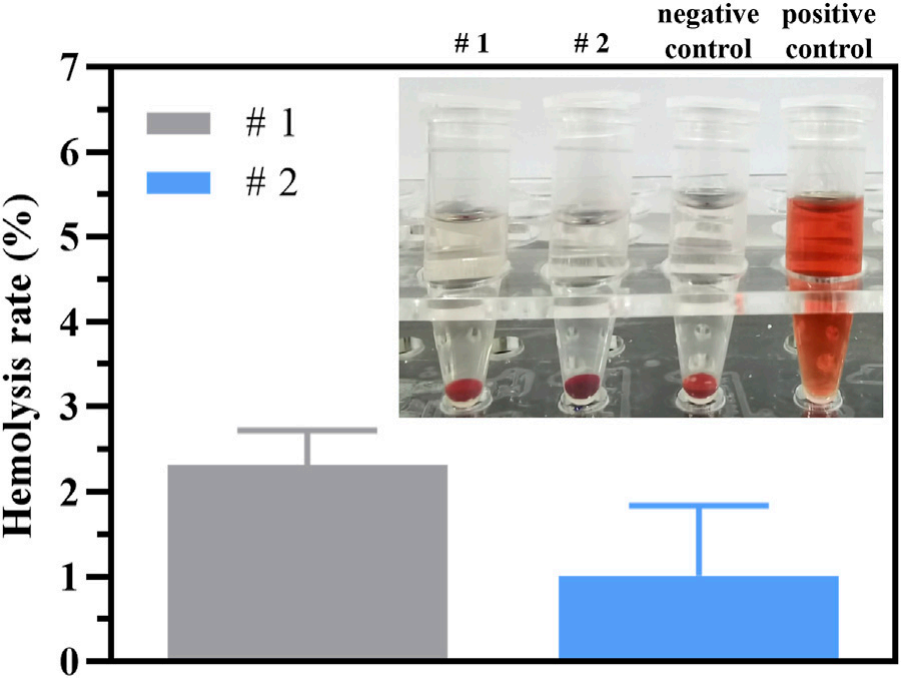
Hemolysis rate of pTi (#1) and Ti-Cu@P(DA-co-SBMA) (#2) and images of hemolysis test of negative control, positive control, pTi (#1) and Ti-Cu@P(DA-co-SBMA) (#2).

Cu^2+^ is toxic, so it is necessary to evaluate the release rate of Cu^2+^ and toxicity of implant materials containing copper. In this section, HUVECs and RBCs were used to assess the toxicity of the coatings. As shown in the fluorescent images (Figures 8A, C), the cells survived in large numbers in each group after 24 h and 48 h of incubation with the samples, indicating negligible cytotoxicity of the coatings. The statistical results of the CCK-8 experiments (Figures 8B, D) show that there were essentially no significant differences in the relative viability of the cells in each group after 24 h and 48 h of incubation. These indicated that the coatings do not have a obvious inhibiting effect on cell proliferation and possess excellent biocompatibility. Some researches reported that the excessive Cu^2+^ may cause erythrocyte hemolysis (Liu et al., 2012; Tong et al., 2020), so it is necessary to evaluate the hemolysis performance of the coating. Figure 9 shows the result of hemolysis test. The hemolysis rate of the original pTi and the final Ti-Cu@P(DA-coSBMA) was equivalent and lower than 5%, which meets the requirements of medical materials for hemolysis rate, indicating that the coating and the original substrate have good hemocompatibility. This can be attributed to the formation of MPNs between copper ions and catechol groups, which makes copper ions more firmly loaded on PDA, avoids the rapid release of copper ions in a short term (Supplementary Figure S7), and ensures the low toxicity and long-term antibacterial ability of the coating. Moreover, the hemolysis rate of Ti-Cu@P(DA-co-SBMA) was less than 2% and could be classified as the nonhemolytic material. This could be ascribed to the good hemocompatibility of PDA and PSBMA.

## 4 Conclusion

In summary, a bio-coating on titanium surface were prepared *via* one-step co-deposition of PDA and PSBMA initiated by CuSO_4_. Meanwhile, the possible reaction mechanism of the co-deposition process reported by previous studies was verified by experiments. The presence of PDA and PSBMA can endow the surface of titanium with excellent hydrophilicity, which is beneficial to reduce the adhesion of thrombocytes and improve biocompatibility. CuSO_4_ can significantly accelerate the oxidation and polymerization of PDA and improve the co-deposition efficiency of PDA and PSBMA. Moreover, Cu^2+^ can form MPNs with the amine and imine groups of PDA, thus improving the stability of the coating and imparting strong antibacterial activity to the surface of titanium. The toxicity test results indicated that the coating has good biocompatibility. This work provides an effective method to address the infection and biocompatibility problems that patients may face after the implantation of artificial hearts. However, the stability of the coating over a longer periods of time (up to several months) needs to be further explored. Additionally, researches on composite antibacterial agents, prothrombin time and activated partial thromboplastin time are meaningful, which are also parts of our future plans.

## Data Availability

The raw data supporting the conclusion of this article will be made available by the authors, without undue reservation.
