# Supplementary material for: Zwitterionic coating assisted by dopamine with metal-phenolic networks loaded on titanium with improved biocompatibility and antibacterial property for artificial heart
Source: Front Bioeng Biotechnol. 2023 Apr 17;11:1167340. doi: 10.3389/fbioe.2023.1167340 (PMC10150318; doi:10.3389/fbioe.2023.1167340)
Supplement: Supplementary file 1 [file DataSheet1.docx]

Supplementary Material

Zwitterionic coating assisted by dopamine with metal-phenolic networks loaded on titanium with improved biocompatibility and antibacterial property for artificial heart

Lingwei Meng, Chuangxin Huang, Xin Liu, Hongyi Qu^*^, Qiuliang Wang^*^

*** Correspondence:** Hongyi Qu: [quhongyi@gia.cas.cn](mailto:quhongyi@gia.cas.cn)

Qiuliang Wang: [qiuliang@gia.cas.cn](mailto:qiuliang@gia.cas.cn)

# Supplementary Data

Luria-Bertani (LB) broth powder, Miller (A507002, FMB grade) and agar (A505255-0250, reagent grade) were purchased from Sangon Biotech Co., Ltd. (Shanghai, China). Pancreatin (BL512A) was obtained from Biosharp (Beijing, China). LIVE/DEAD cell staining kit (BB-4126) was purchased from BestBio (Shanghai, China). Cell counting kit-8 (CCK-8, IV08-100) was obtained from Invigentech (USA). 4% paraformaldehyde fix solution was purchased from Beyotime Biotech. Inc. (Shanghai, China). Ultrapure water was obtained from a Milli-Q system (Millipore, USA). The other chemical and biological reagents not mentioned were obtained from Shanghai Aladdin Biochemical Technology Co., Ltd. (Shanghai, China) and Shiyanjia Lab, respectively.

# Supplementary Figures


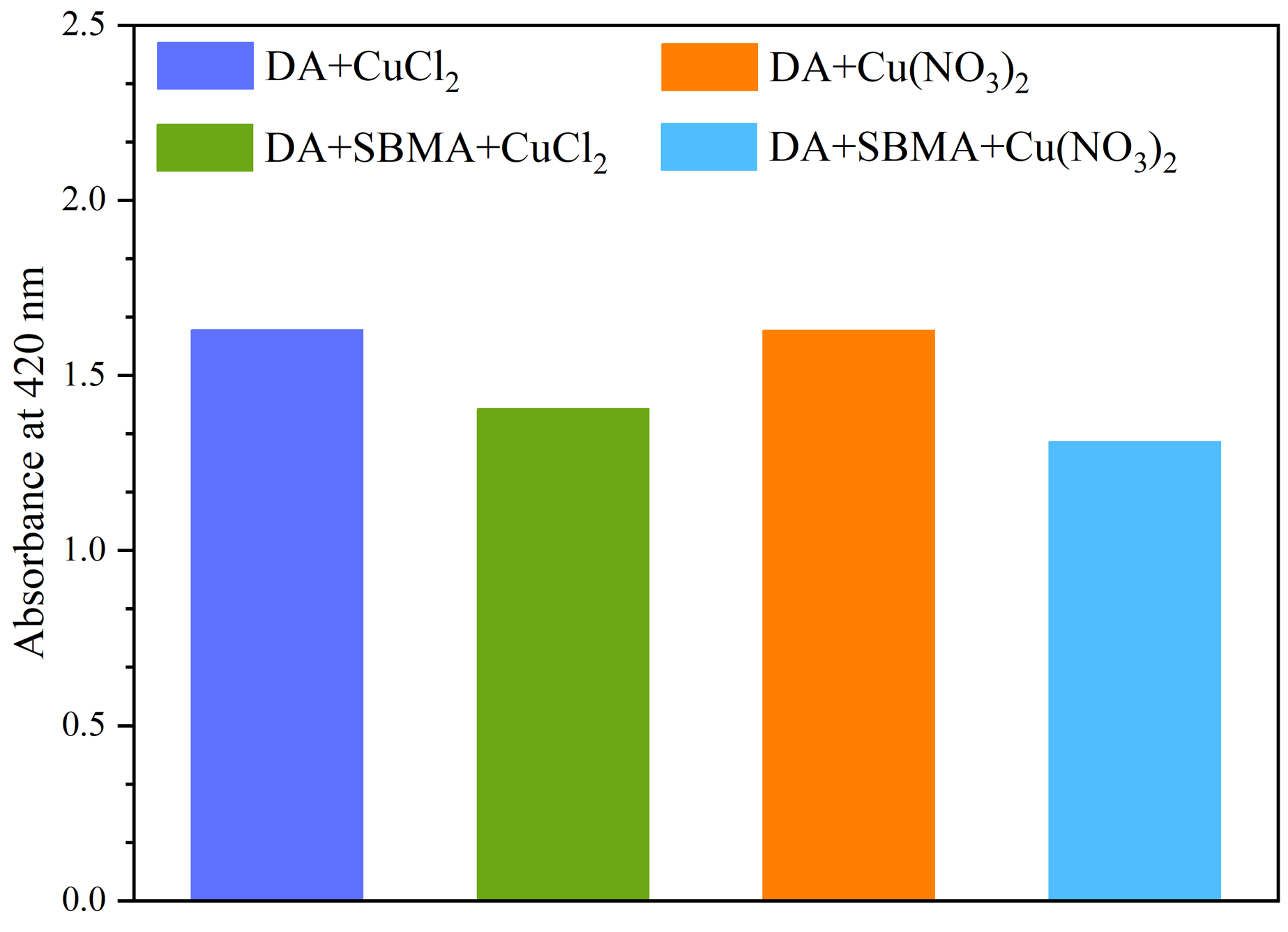


**Supplementary Figure S1.** UV–vis absorbance of different solutions at 420 nm after 30 minutes.


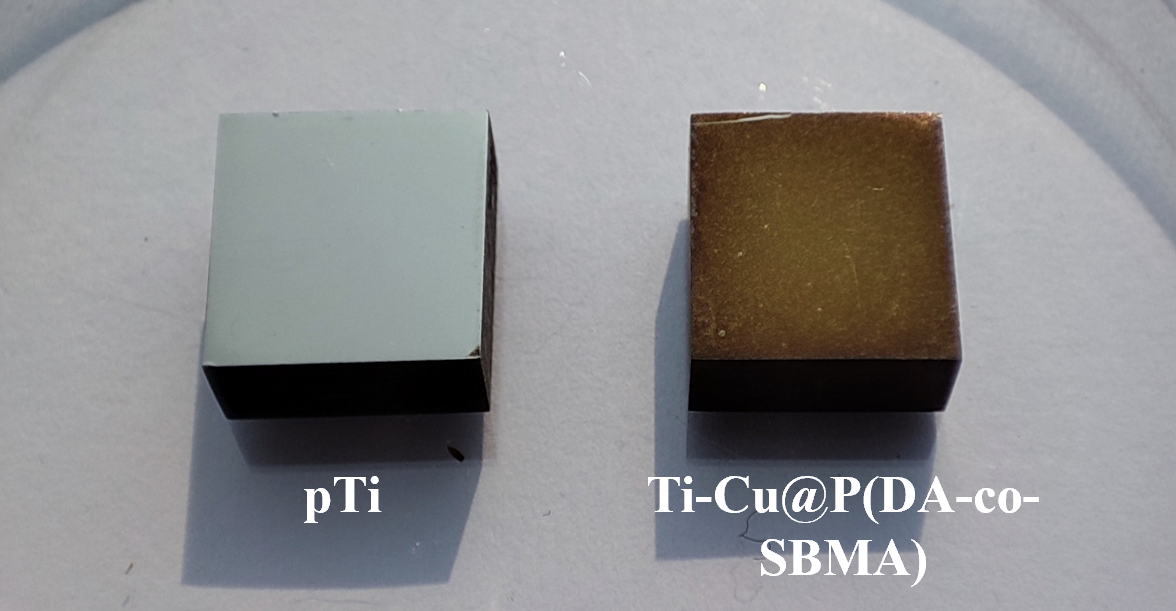


**Supplementary Figure S2.** Photographs of the pTi and Ti-Cu@P(DA-co-SBMA).


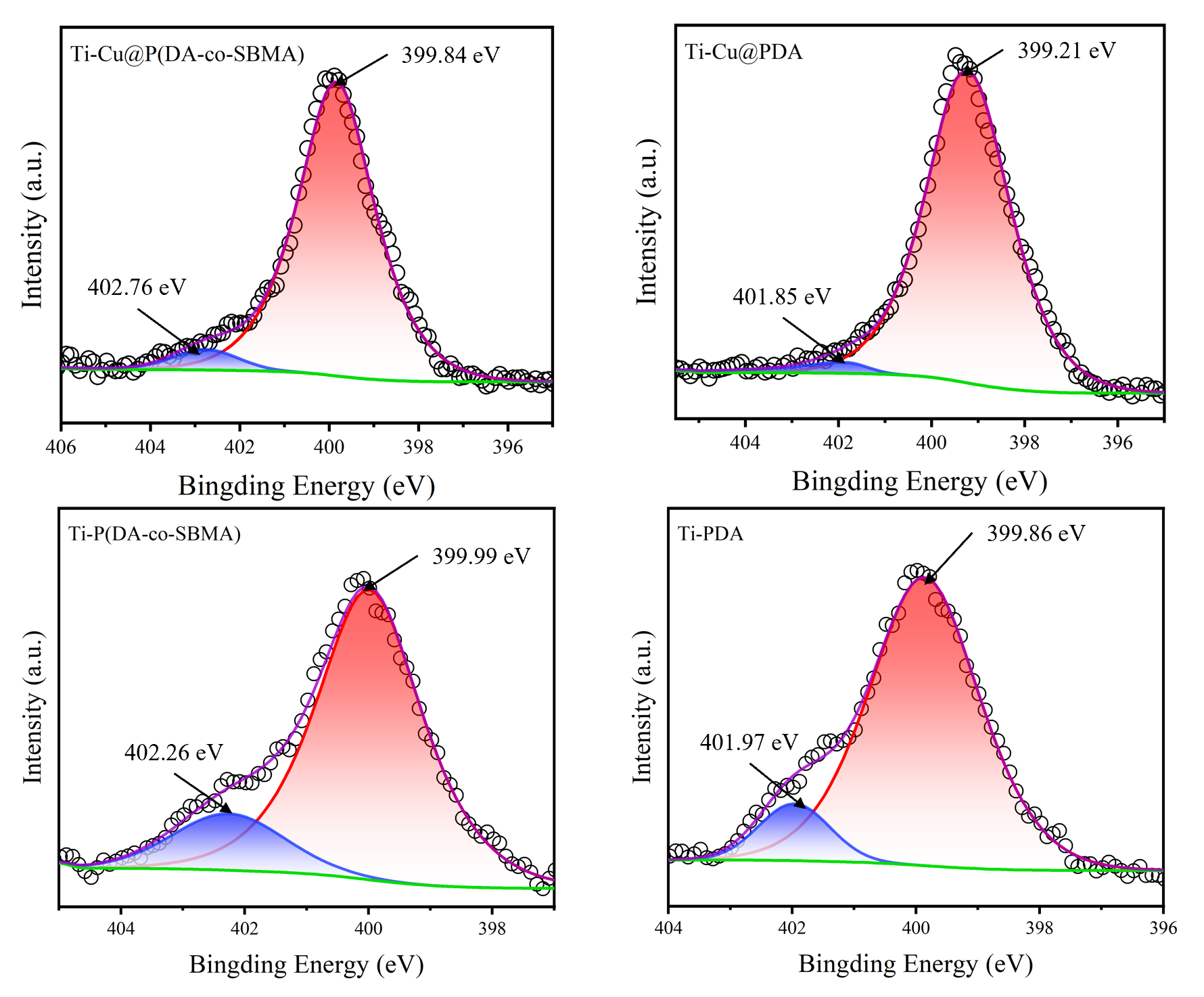


**Supplementary Figure S3.** High-resolution N 1s spectra of the Ti-PDA, Ti-P(DA-co-SBMA), Ti-Cu@PDA and Ti-Cu@P(DA-co-SBMA).

**
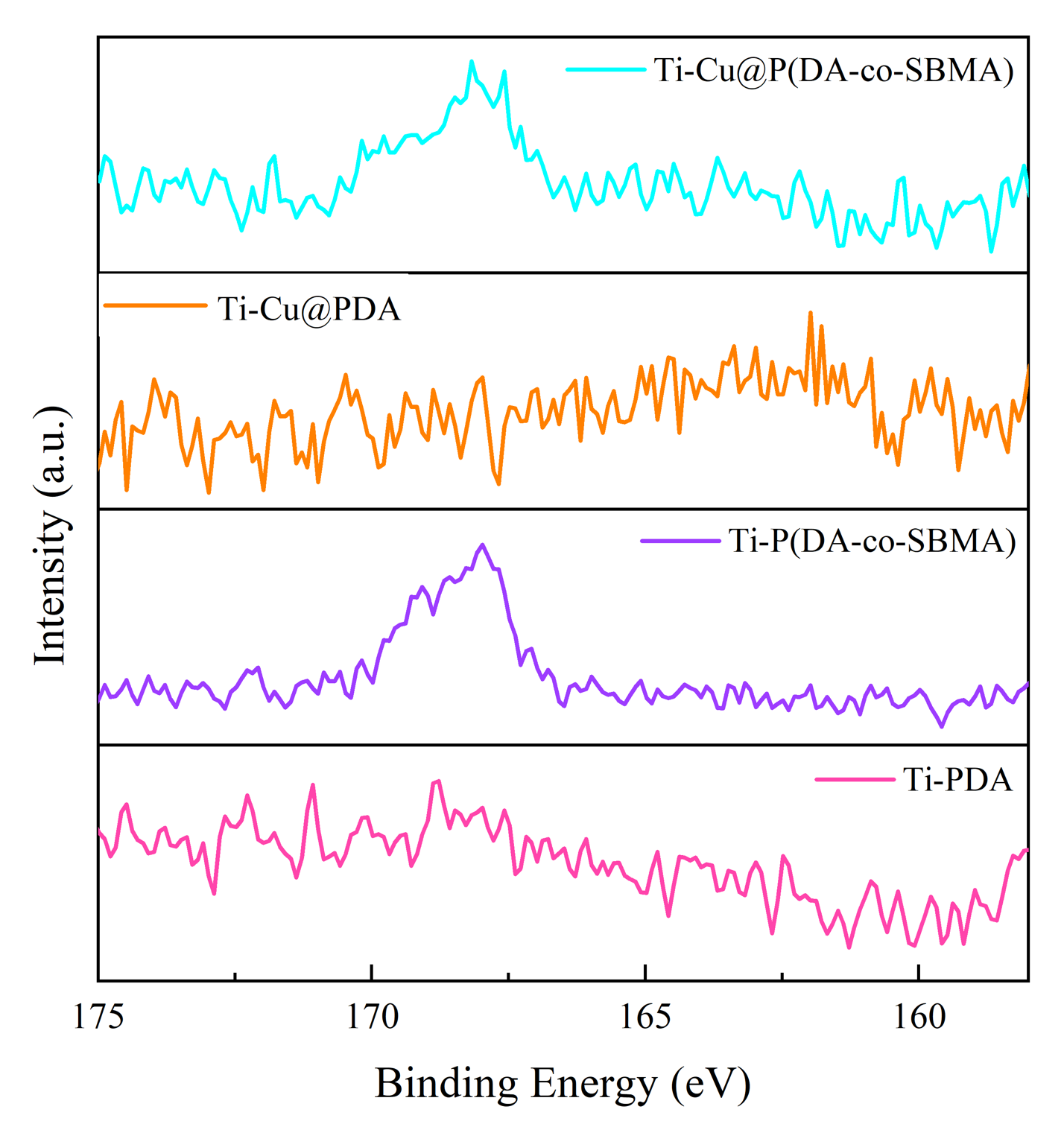
**

**Supplementary Figure S4.** High-resolution S 2p spectra of the Ti-PDA, Ti-P(DA-co-SBMA), Ti-Cu@PDA and Ti-Cu@P(DA-co-SBMA).


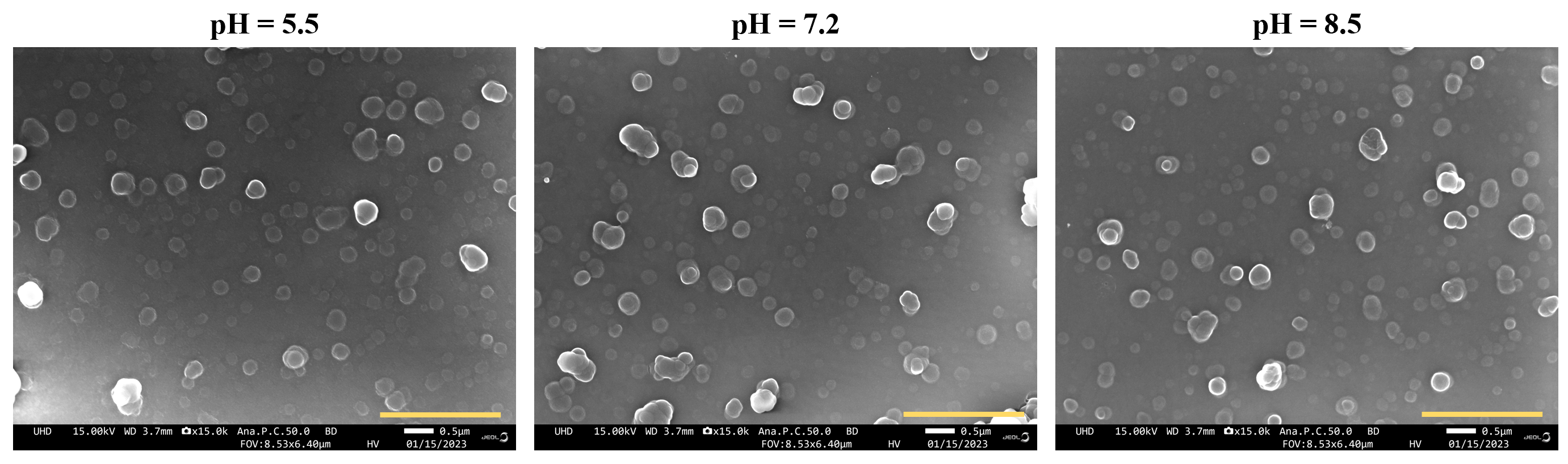


**Supplementary Figure S5.** SEM images of the Ti-Cu@P(DA-co-SBMA) immersed in different PBS buffer solutions after 7 days. Scale bar: 2 μm.


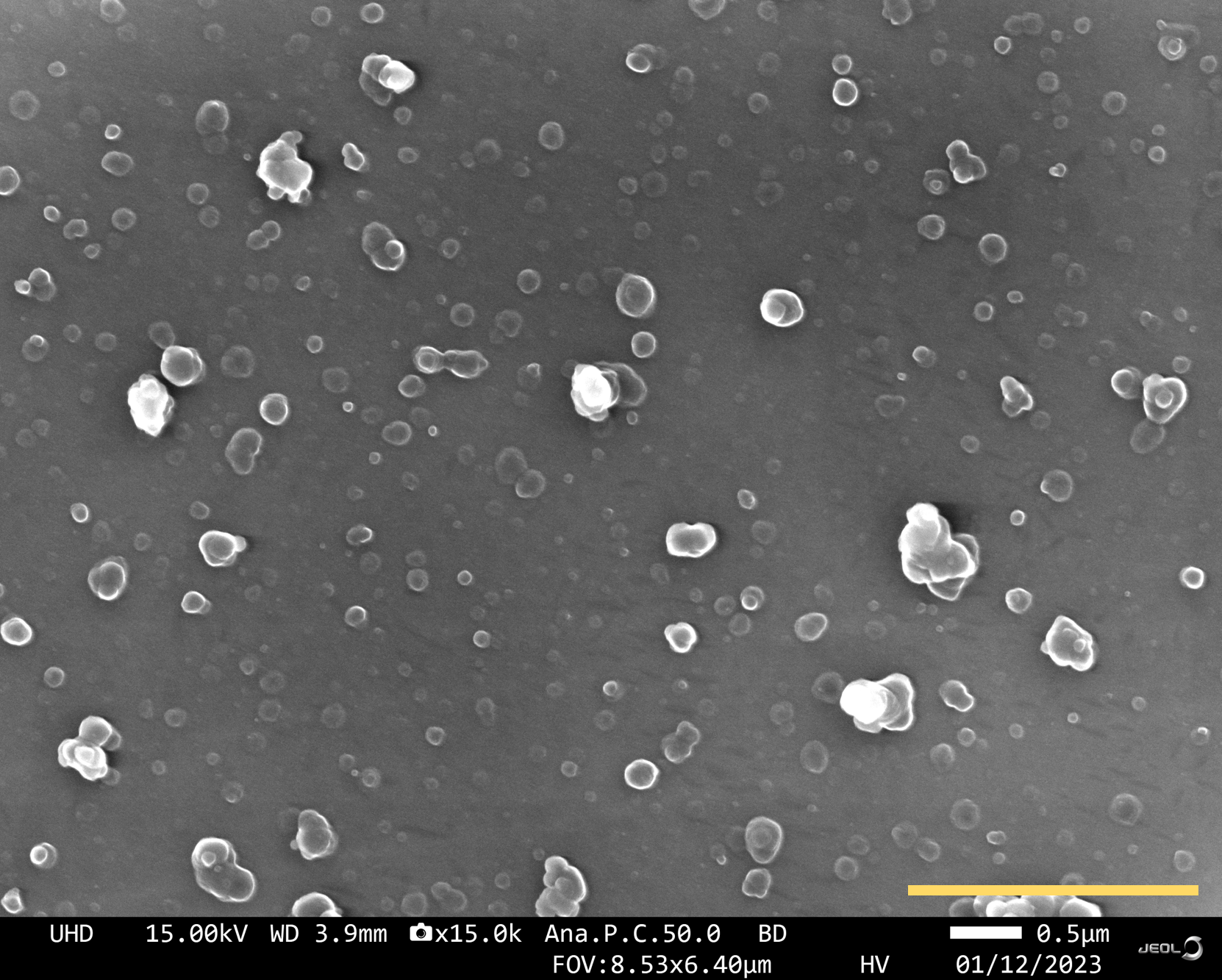


**Supplementary Figure S6.** SEM image of the Ti-Cu@P(DA-co-SBMA) exposed to air after 7 days. Scale bar: 2 μm.


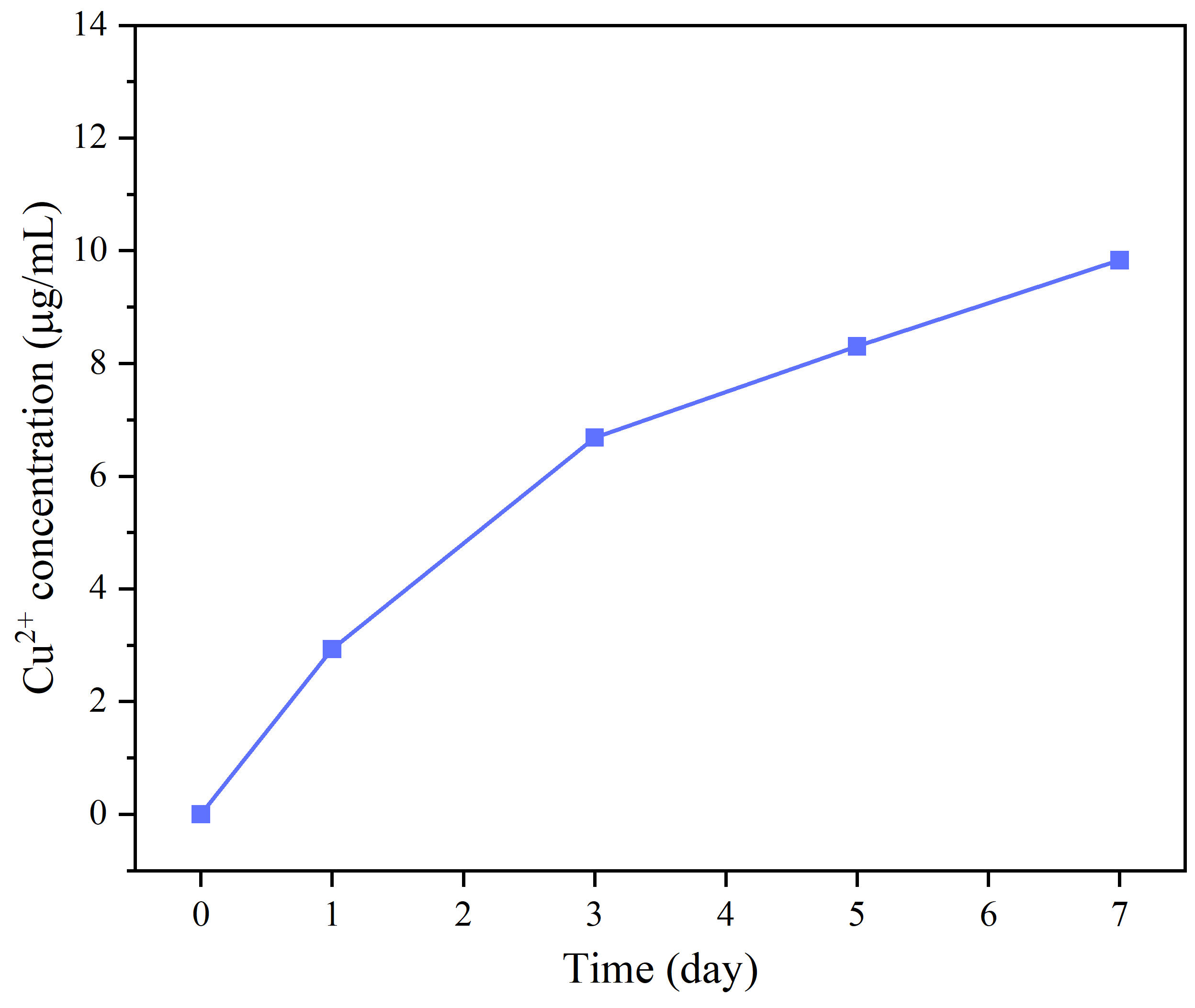


**Supplementary Figure S7.** Cumulative released concentration of Cu^2+^ from Ti-Cu@P(DA-co-SBMA) in 10 mL of PBS.
